# Supplementary figures and images for: Comparative analysis of whole flower transcriptomes in the Zingiberales
Source: PeerJ. 2018 Aug 24;6:e5490. doi: 10.7717/peerj.5490 (PMC6110254; doi:10.7717/peerj.5490)

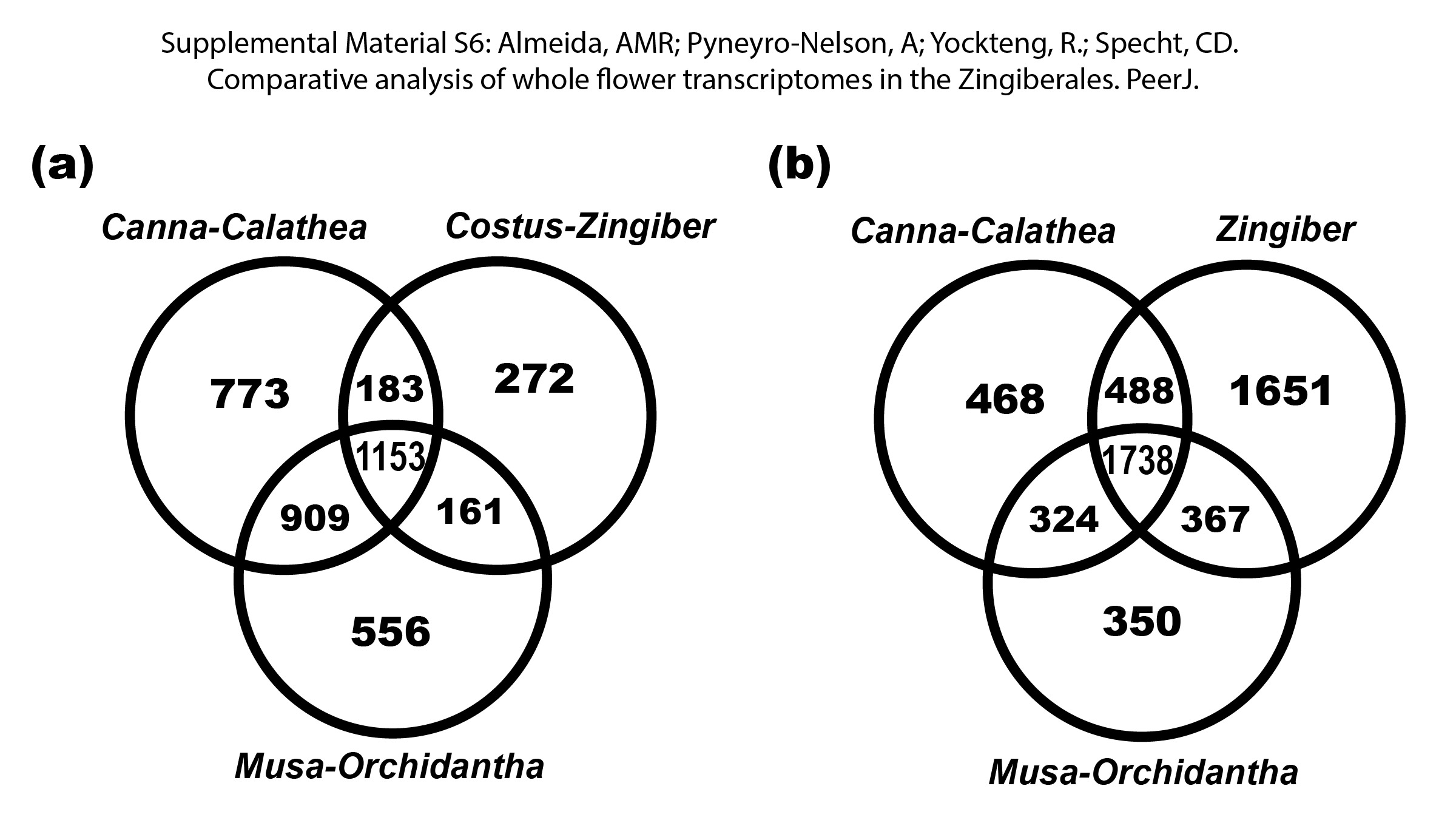

Supplement: Supplemental Information 6 [file peerj-06-5490-s006.zip › Supplemental Material S6.jpg]
